# Nursing faculty development program for digital teaching competence

**DOI:** 10.1186/s12909-024-05453-8

**Published:** 2024-05-08

**Authors:** Soonyoung Shon, Hyunsook Shin, Dahae Rim, Hyejin Jeon

**Affiliations:** 1 Department of Nursing, Keimyung University, Daegu, Republic of Korea; 2 College of Nursing Science, Kyung Hee University, 26 Kyungheedaero, Dongdaemungu, Seoul, 02447 Republic of Korea

**Keywords:** Nurse education, Simulation, Nursing practice, Digital teaching competencies, Distance education

## Abstract

**Background:**

Nursing faculties need to develop digital competencies to effectively use information, communication, and technology-based nursing education.

**Purpose:**

The study aimed to develop and apply a theory-guided faculty development program on digital teaching competencies.

**Methods:**

A faculty development program was developed. Between March and April 2020, three five-hour web workshops participated by ninety-three faculty members were held. The program was assessed via mixed methods, combining satisfaction surveys post-workshop with content analysis of open-ended questionnaires to gauge participant evaluation of program content and learning experience.

**Results:**

Participants were highly satisfied with the program contents and their opportunity for integrating digital technology into education and improving faculty proficiency in digital teaching technology.

**Conclusions:**

The program provides faculties with the self-confidence and essential skills to teach students using information, communication, and technology-based nursing education by enhancing their digital teaching competencies. It is critical to integrate both digital proficiency and nursing practice education.

## Background

Technology significantly influences teaching and learning environments. Rapidly evolving digital technologies offer opportunities to continue and enhance professional training traditionally reserved for real practice settings such as medical institutions [1]. Digital technologies include mobile, virtual, and online platforms, enable nurse faculties to teach students clinical practicum. These teaching situations have been accelerated and emphasized by pandemic disaster such as the Middle East respiratory syndrome coronavirus (MERS), Severe Acute Respiratory Syndrome (SARS), Ebola, and current coronavirus disease (COVID-19) when clinical practicums could not be provided in hospitals and the community, and Hao et al. reported that is effective approach in the medical and nursing education [2]. Specifically, higher education sector was severely affected by the COVID-19 pandemic, leading faculties to hastily convert their curriculum to an online format. However, there are many challenges associated with this transition, including academics’ home-office infrastructure and digital teaching competence as well as learners’ access to the online teaching environment [3].

Recently, professional education in fields such as nursing education, which traditionally rely on methods such as preceptorship, mentorship, and clinical shadowing, has shifted to online platforms [4]. Thus, there is a heightened emphasis on enhancing nursing faculties’ pedagogical digital competence (PDC) to effectively teach students, reflecting the urgent need to develop faculties’ digital competence for virtual and online education. However, despite this necessity, most faculty development programs provide offline programs or limited webinar formats. A proficiency in digital teaching competency is imperative, particularly in light of the current pandemic and the imperative to maintain social distancing mandates [5].

General teaching competencies in nursing education encompass leadership ability, problem solving ability, educational intelligence, general teaching proficiency, and clinical nursing skills [6]. However, with the recent development of digital technology, there has been a growing emphasis on digital teaching competence, including Information and Communication Technology (ICT) utilization. This competence is often referred to interchangeably as pedagogical digital competence (PDC) or digital competence of educators.

Previously, the United Nations Educational, Scientific, and Cultural Organisation (UNESCO) suggested a new paradigm in teaching competence using ICT and developed a module to enhance teachers’ ICT competence that consisted of understanding ICT in education, curriculum and assessment, pedagogy, ICT, organization and administration, and professional learning [7]. UNESCO introduced a dimension in pedagogical skills and competencies known as PDC, which encompasses knowledge, skills, attitudes, approaches to technology, learning theory, subject, context, and their relationships. PDC refers to the ability to consistently apply attitudes, knowledge, and skills required to plan, conduct, evaluate, and revise ICT-supported teaching on an ongoing basis [8]. Likewise, the framework for Digital Competence of Educators published by the European Commission’s Joint Research Centre focuses on how digital technologies can enhance and innovate educators’ professional competencies, pedagogic competencies, and learners’ competencies [9].

**Fig. 1:**
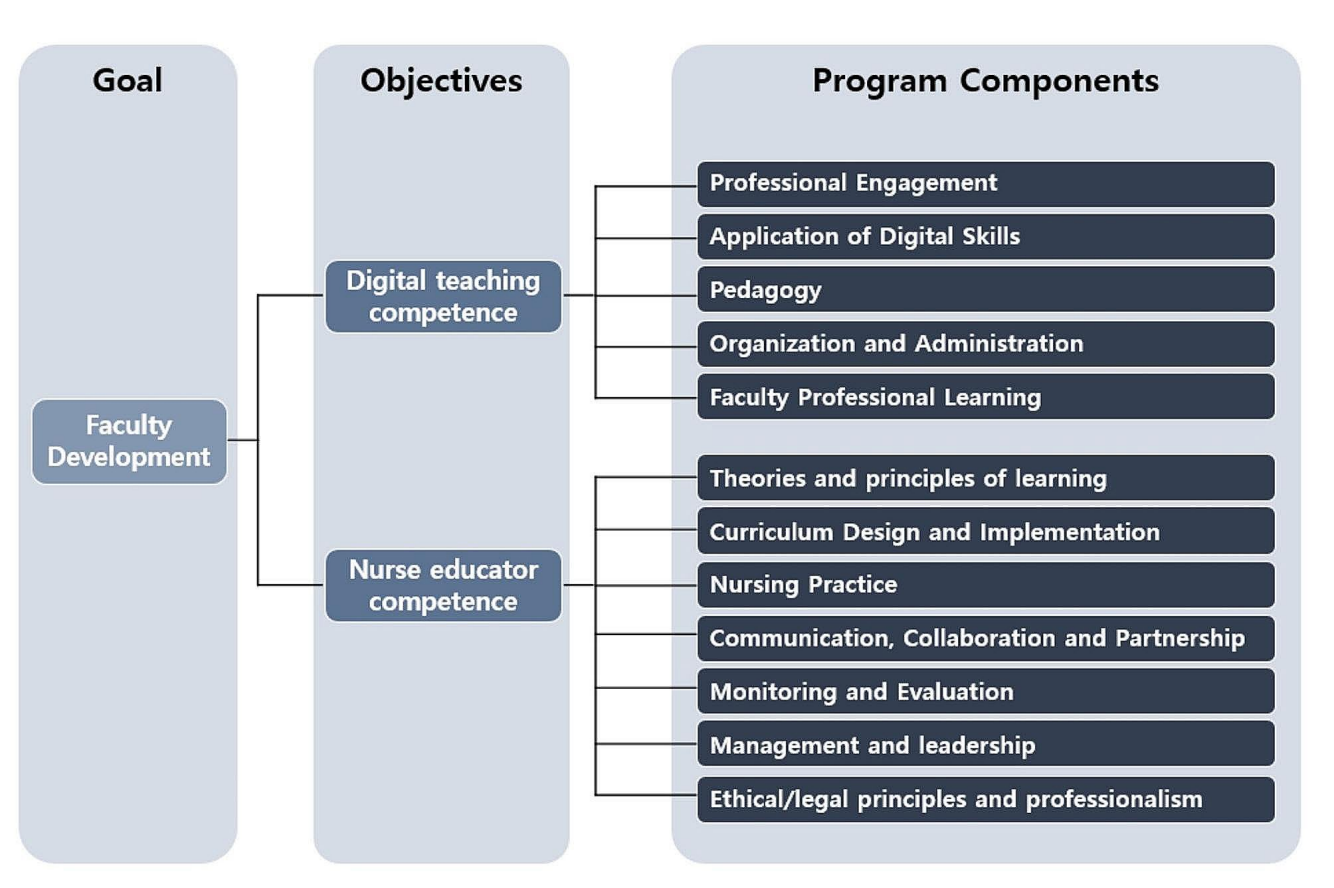
Conceptual framework used to develop the web training program to enhance digital teaching competence

A nursing faculty development program for digital teaching competency was developed in respond to an urgent need for virtual and online practicum opportunities due to COVID-19. Academic leadership recognized nursing faculties were more prepared for virtual/online education, especially regarding practicum strategies in a virtual and online environment. The goal of the program was to provide a training course for nursing faculties to become familiar with the digital teaching environment and to apply appropriate teaching strategies for this setting. A multi-user virtual environment simulation, a virtual community program, and a train-the-trainer (TOT) program offered as modules and practice opportunities, designed as a five-hour workshop. It includes general digital teaching and professional training for introducing online resource and improving utilization ability. We developed the training program utilizing a conceptual framework created by combining key elements from PDC and nurse educator core competencies. The framework was useful for designing training opportunities facilitated by virtual and online environments. The components of the program included pedagogy competence, digital resource mobilization, teaching and learning strategies, assessment abilities, and strategies for empowering learners. Figure 1 illustrates the conceptual framework used to develop the faculty development program aimed at enhancing their digital teaching competence.

### Aim of the study

The purpose of this study was to introduce the nursing faculty development program for digital teaching competency and to explore nursing faculties experience of ICT competence enhancing trough workshop.

## Methods

### Design

We developed the program and evaluated it using mixed-method. To determine how participants evaluated the program content and understood the learning experience, a descriptive evaluation was conducted through satisfaction survey after the workshop and session. Additionally, content analysis was performed using an open-ended questionnaire of participant’s experience.

### Workshop process

**Fig. 2:**
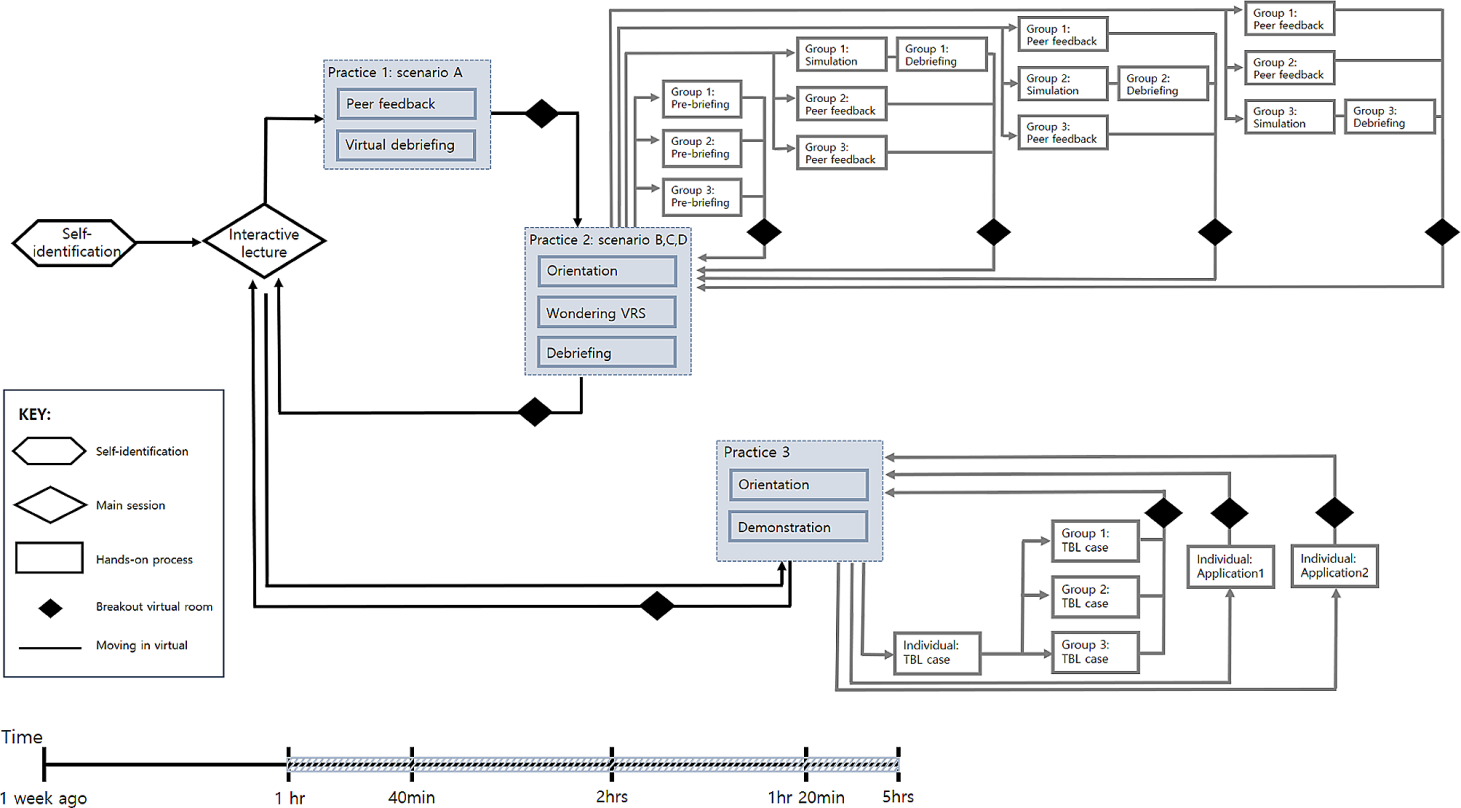
Flow diagram of process followed in the web training workshop Note: VRS: Virtual Reality Simulation; TBL: Team-Based Learning

The workshop was developed based on PDC and nurse educator core competencies. The strategies employed in the workshop included self-identification, interactive lectures, simulation practice, and self-evaluation. The content focused on understanding the virtual space, applicable platforms and resources, hands-on practice, and the implementation of a nursing practice curriculum. We conducted the workshop three times between March to April 2020. Each workshop was presented to a group of faculties with three facilitators and lasted approximately five hours (Fig. 2). The first workshop was held on March 12, 2020, with 28 faculties from 28 different colleges of nursing, the second was held on April 3, 2020, with 33 faculties, and the third was held on April 23, 2020, with 32 faculties from different universities. The five hours covered content delivery and opportunity for participants to practice learning skills virtually. After each workshop, the team (head, two facilitators, one teaching assistant, and one technology assistant) held an online meeting to discuss what was successful and what to improve. All the facilitators were responsible for posting on discussion boards. The following statements were the suggested educational objectives of the workshop.

By the end of the nursing faculty development program, participants will be able to:

- Describe the theoretical framework of virtual and online simulation.
- Explain major resources used for virtual/online simulation.
- Develop how to compose and manage instructional content for virtual/online simulation.
- Demonstrate how a virtual/online simulation should be performed with students.

Facilitator and learner guides were provided to prepare for the workshop. Before the program, we requested participants to download the required software applications including Second life, Zoom, and online sharing applications such as Google Docs, sheets, and sharing drive. This allowed workshop participants some time to become familiar with their digital devices and the new applications. Additionally, we provided digital versions of worksheets and tools for participants to use during the workshop.

Participants were asked to assess their current practicum curriculum and coursework. They were then asked to design and plan their practicum courses using virtual/online strategies learned during the workshop. In a pre-session survey, participants were asked to provide basic demographic information such as their age, teaching experience, and their use of technology. Participants were asked in a post-session questionnaire to evaluate the format, materials, and resources of the program and to describe their learning experience.

### Participants

There were 93 nursing faculty members from 74 universities who participated in one of the three online workshops conducted between March and April 2020. On average participants’ age were in the mid-40s. The average teaching experience was 11.46(± 8.80) years. Two thirds of the professors specialized in paediatric nursing and a third specialized in women’s health nursing. In the brief pre-session survey, respondents reported that they use on average of 3.00(± 0.88) pieces of Information Technology (IT) equipment; the majority used a laptop followed by smartphones, desktop computers, and smartpads, in decreasing order. More than half of the respondents reported that it is easier to use technology at home than in their classrooms, but the difference was small. Of the workshop participants, the most said it was their first experience of such an online workshop.

### Instruments and Data collection

We used a program satisfaction questionnaire and open-ended questions for evaluating the implemented program using Google survey that the research team developed according to the previous study related learning satisfaction and continuing learning intention [10]. The program satisfaction survey questionnaire consisted of three categories with a total of 17 items, and there were four open-ended questions. Participants were asked to indicate on a 5-point Likert scale (where 1 = Strongly disagree and 5 = Strongly agree), how satisfied they were with the program. Perceptions of the appropriateness of the workshop’s duration was indicated by length of time qualifiers (1 = very short, 3 = proper, and 5 = very long). We asked specific questions about general satisfaction, Content (Overall, Participatory learning), and Management. Participants provided responses to open-ended questions about improving the program: ‘What aspects of the program do you think need to be improved?’, ‘Do you have any topics or content that you want to learn more about in this program?’, and ‘Do you have any other comments?’ Participants were also asked to rate their agreement/disagreement with the statement ‘The workshop strengthened the proficiency for establishing nursing practicum operation strategies in virtual/online spaces’ using a 5-point Likert scale ranging from strongly disagree [1] to strongly agree [5]. The survey questions were validated by nursing facilities with education experiences. The Cronbach’s alpha was 0.92 in this study. The open-ended questions allowed participants to elaborate on their responses to closed-ended questions. The contents of the discussion posted on the discussion board were also used for analysis. We requested satisfaction surveys from 93 participants in the training, of whom 66 participated, resulting in a response rate of 71%.

### Ethical considerations

Following institutional policy and procedure, permission to distribute the questionnaires was obtained from the Academies of Nursing. In the cover letter, participants were informed of the study purpose and their rights of voluntary participation and confidentiality. Informed consent was obtained from all participants who voluntarily agreed to participate before the participation.

### Data analysis

Descriptive statistics were used to summarize and describe the participants’ quantitative ratings of the program. Content analysis was used to analyse the data from the open-ended questions in the post-session evaluations. The components of the conceptual framework of the nursing faculty development program for digital teaching competency were used to guide the analysis and categorize the data. Participants’ comments on the program were classified into each component of the framework. The analysis of the data was based on analytical framework. The researchers read participants comments and identified the theme. Three investigators independently reviewed the comments, placed them into respective category, identified them, and providing rationales for the selection. When roughly one third of the comments were categorized, the researchers discussed the categorizations, shared categories, and reconciled differences in their categorization schemes. Through these discussions, categories were combined, divided, or renamed, or the rules for inclusion were rewritten. The researchers then proceeded with independent categorization, utilizing newly-negotiated scheme from the analytical framework. Two more of these kinds of group discussions took place. Through this process, a classification scheme of digital teaching competence in nursing education was constructed. Inter-rater agreement was averaged, ranging from 70 to 90%. The analytical framework is presented in Table 1 with detailed descriptions of each component.

**Table 1:** Conceptual framework of the web-based faculty development program

Domain	Component	Description
Digital teaching competence	Professional Engagement	Aware of how ICT might be aligned with education priorities within the educational policies. Understanding digital devices applicable to education.
	Application of Digital Skills	Determine tools and devices appropriate for the task at hand. Identify the function of digital tools to reinforce and enhance learning.
	Pedagogy	Acquire ICT skills to support effective teaching and learning methods. Planning, designing, and arranging the use of digital technologies. Integrate digital resources and methods (to promote collaborative and self-regulated learning processes, learner-centred processes and activities)
	Organisation and Administration	Organising the physical environment in a virtual space. Create an environment that facilitates effective learning outside the classroom and in virtual environments.
	Faculty Professional Learning	Digital Continuous Professional Development. Participate in lifelong professional development.
Nurse educator competence	Theories and principles of learning	Understanding the conceptual and theoretical foundations and principles. Knowledge of curriculum development in nursing practice education.
	Curriculum Design and Implementation	Designing a nursing practice curriculum to support the needs of nursing practice in context. Create and maintain a safe environment for learning about nursing practice Engage learners with appropriate information technology. Formulating evaluation tools for learners’ experience.
	Nursing Practice	Practice learners with evidence-based approach and current knowledge. Planning a variety of learning activities for learners that promote creativity and innovation in nursing practice and health care environments.
	Communication, Collaboration and Partnership	Facilitate and foster learners’ teamwork and collaboration Foster intercultural and interdisciplinary competence.
	Monitoring and Evaluation	Foster learners’ self-assessment skills and reflection on teaching and learning activities. Use a variety of assessment tools and methods.
	Management and leadership	Researching effective and efficient human and financial resource management. Use a variety of advocacy strategies to promote nursing education and practice.
	Ethical/legal principles and professionalism	Facilitate professionalisation as a pre-registration nurse for learners by fostering self-reflection, personal goal setting, and socialisation

ICT: Information and Communication Technology

## Results

### Evaluation of the program

Sixty-six (71.0%) participants responded to the course evaluation questionnaire for the program. Table 2 presents the descriptive results of the evaluation ratings pertaining to participant’ satisfaction with the program. The average satisfaction score of the program was quite high. Respondents indicated that the workshop met their interests, exceeded their previous expectations, and that they would recommend the workshop to others. Respondents were more than satisfied with the overall workshop content and were generally satisfied with the participatory program’s content. In particular, they indicated that the workshop content met their goals and objectives and that they were able to achieve them. They also expressed above-average satisfaction with the overall operation of the nursing faculty development program. Respondents, however, reported in the open-ended questions the need for more practicing time and opportunities as well as improved management.

**Table 2:** Descriptive results of participants’ satisfaction with the web-based workshop

Category	Subcategory	Item		M(± SD)	*n*(%)
		The workshop met the interest and expectations of the prior participation.		4.65(± 0.54)	
General satisfaction		I would recommend the workshop to others.		4.74(± 0.53)	
Contents	Overall	achieved proposed goals and objectives.		4.70(± 0.50)	
		were relevant to goals and objectives		4.77(± 0.46)	
		The level of detail contents was appropriate		4.39(± 0.76)	
		provided ample opportunities for discussion and practice.		4.14(± 0.91)	
	The Participatory Learning	achieved presented goals and objectives of the workshop.		4.59(± 0.61)	
		was relevant to goals and objectives of overall workshop		4.64(± 0.54)	
		was operated with appropriate distribution of team members (by number)		4.33(± 0.83)	
		was operated with appropriate time for team-working		3.94(± 0.93)	
		Provided ample opportunities for discussion and practice.		4.14(± 0.86)	
Operation	Overall	Overall Operation was satisfactory.		4.64(± 0.60)	
	Composition	Presentation lectures and participatory learning programs were properly organised.		4.27(± 0.69)	
	Time	The duration of the workshop was appropriate	Short		10(15.2)
			Proper		41(62.1)
			Long		10(15.2)
			Very long		5(7.6)
	Environment	The Workshop operating environment was appropriate.		4.62(± 0.60)	
	Administration	Administrative procedures were easily carried out. (e.g. registration, operation, etc.)		4.55(± 0.64)	
		Administrative procedures were satisfactory. (e.g. registration, operation, etc.)		4.65(± 0.57)	
overall				4.50(± 0.25)	

### Faculty proficiency in Digital Teaching Technology

Participants strongly agreed that they improved their proficiency in digital teaching technology for nursing practicum operation. Table 3 presents the summarized descriptive results from participants’ open-ended responses as they relate to PDC in the analytical framework.

The theme ‘Faculty proficiency in digital teaching technology’ dealt with the categories *Engagement, Application of digital technology to curriculum, Pedagogy, Management of learning environment, and Faculty professional learning*. The most significant categories were *Application of digital technology to curriculum* and *Engagement*. In the category of *Engagemen*t, participants demonstrated ‘Awareness’ of the digital technologies in education and even reported in-depth ‘Understanding’. Participants also highlighted that they could provide a ‘Demonstration of supporting digital technology in the curriculum’ and felt they were ‘Achieve the curriculum goal through digital technology’, ‘Identifying digital resources’, and could ‘Select appropriate digital resources’, in the category of *Application of digital technology to the curriculum*. The category of *Pedagogy* contained ‘Educational technology integration’ and ‘Implementing collaborative learning’, and *Management of learning environment* included the subcategory of ‘Extended Learning Environment’. In addition, under the category of *Faculty professional learning*, participants requested for ‘Continuous opportunities for digital professional development’, ‘Detailed strategies for virtual nursing practicum’, and ‘Programs for faculty specialisations’.

**Table 3:** Open-ended responses relating to pedagogical digital competence in the conceptual framework

Category	Subcategory	Illustrative quote	
Engagement	Awareness	*‘I was able to get ideas for operating a nursing practicum using digital resources in the current situation’.*	
	Understanding	*‘In addition to the current difficult situation of non-face-to-face practice, the educational aspect of allowing learners to actively participate in nursing practicum has given us a lot of thoughts and ideas’.*	
		*‘A variety of educational methods and approaches that can be utilized in Web based-lectures’.*	
		*‘It can pursue methodological diversity in teaching in the classroom as well as nursing practicum, which will greatly help improve the quality of future teaching and learning’.*	
Application of Digital Technology to Curriculum	Demonstration of Supporting Digital Technology in the Curriculum.	*‘(I was able to get) information and instructions on how to use multiple methods for online classes and practicum (e.g. PC, online, mobile, etc.)’.*	
	Achieving curriculum goals through Digital Technology	*‘I was able to get information on the methodology of nursing practicum in the classroom and online’.*	
		*‘(I was able to learn) strategies by utilising online spaces in the situation of non-face-to-face classes and practicum’.*	
		*‘I can try to operate virtual nursing practicum’.*	
	Identification of Digital resources	*‘I was provided with information about various Web materials’.*	
		*‘(I have learned that I) can apply software that I can implement with the nursing practicum’.*	
	Selection of appropriate Digital resources	*‘Vital sign training using online sources’.*	
		*‘Simulation practicum using virtual reality environment’.*	
Pedagogy	Educational Technology Integration	*‘Applying the Web workshop program for nursing practicum’.*	
	Implementing Collaborative Learning	*‘Simulation practicum using virtual reality environment’.*	
		*‘(I will be able to) apply (digital technology) to feedback in the simulation practicum’.*	
Management of Learning Environment	Extended Learning Environment	*‘(I was able to learn) strategies by utilising online spaces in the situation of non-face-to-face classes and practicum’.*	
		*‘I’ve taken some direction on how to teach students online’.*	
Faculty Professional Learning	Continuous Opportunities for Digital Professional Development	*‘Through regular seminars of academic societies, I would like to share and study virtual practical training methods and good examples’.*	
	Detailed strategies for virtual nursing practicum	*‘Objective Participation of Students in Virtual Simulation and Debriefing’.*	
		*‘(I want to learn) TBL-based online case studies and feedback/or instruction methods and content, in detail’.*	
	Programs for Faculty specialisations	*‘(I) want to share women’s health content that can be applied to clinical practicum using Web or mobile resources’.*	

### Digital Technology Integration for Nursing Practice Education

The results of the open-ended responses to integrating digital technology in nursing practice training as it relates to the nurse educator core competency in the conceptual framework is presented in Table 4. This theme contained seven categories, namely *Theories and principles of learning*, *Curriculum design and implementation*, *Nursing practice*, *Communication, collaboration and partnership*, *Monitoring and evaluation*, *Management and leadership*, and *Ethical/legal principles and professionalism*. In the analysis, the last two categories were combined into a category of *leadership and professionalism.*

The most significant categories were *Nursing practice* and *Communication, collaboration and partnership.* Participants reported that they could integrate digital technology with nursing practice, especially ‘Skill Training in a Virtual Environment’ and ‘Online Case-based Learning’. They also acknowledged that the subcategories of ‘Effective Communication Skills’, ‘Collaboration for professional learning’, and ‘Sharing online/virtual teaching content for resource-limited environments’ could improve nursing practice education by utilising digital technology. The response to the category of *Curriculum design and implementation* to digital technology integration with nursing practicum was also prominent. This category included ‘Designing a nursing practicum curriculum’ and ‘Creating and modifying digital resources for teaching’. Moreover, the category of *Monitoring and Evaluation* corresponded with the subcategory ‘Utilizing a variety of strategies to assess student learning’, and the category of *Theories and principles of learning* included the subcategory of ‘Applying Theories for Teaching in Online Environments’. Finally, *Leadership and Professionalism* was consistent with the subcategory of *‘*Insisting Urgent Regulation Preparation for Uncertain Situations’.

**Table 4:** Open-ended responses to integrating digital technology in nursing practice training as it relates to the conceptual framework’s nurse educator core competencies

Category	Subcategory	Illustrative quotes
*Theories and principles of learning*	*Applying Theories for Teaching in Online Environments*	*‘I’m willing to use them in my child health assessment section when we try to improve our student critical thinking through online practicum’.*
*Curriculum Design and Implementation*	*Designing a Nursing Practicum Curriculum*	*‘(It can be applied) to organise and operate paediatric nursing practicum using online content’.*
		*‘I was able to get ideas about the configuration of practical content and I think I could apply it’.*
	*Creating and Modifying Digital Resources for Teaching*	*‘(We need) Training for the virtual simulation environment development process’.*
		*‘I would like to know the process or method of creating simulation algorithms for various situations to the point of AI deep learning’.*
*Nursing Practice*	*Skill Training in a Virtual Environment*	*‘Vital sign assessment training using online sources (will be available for online nursing practicum)’.*
		*‘Skill training program using online sources (will be available for online nursing practicum)’.*
		*‘Having hands-on virtual training in XX community (Home visiting) helped prepare for online nursing practicum’.*
		*‘It is likely to replace clinical skills education’.*
	*Online Case-based Learning*	*‘It is likely that a clinical practice case can be provided by establishing a site-friendly clinical site online’.*
		*‘It is thought to be possible to provide students with clinical cases and to carry out Team-based learning’.*
*Communication, Collaboration and Partnership*	*Effective Communication Skills*	*‘It was helpful to perform the simulation feedback through online systems by myself as a learner and I think we can apply it to practical training’.*
		*‘All the processes that experienced real-time communication with multi-users were beneficial’.*
		*‘I think I can apply what I learned today to interact with students in real time’.*
	*Collaboration for Professional Learning*	*‘I hope that we can share the actual data of representative clinical cases in our academic society and use them for education. It would be nice if case-based protocol development or case-based modules could be shared’.*
	*Sharing Online/Virtual Teaching Content for Resource-limited Environment*	*‘It is necessary to share practical strategies that can be applied by institutions that do not have a Virtual learning environment’.*
*Monitoring and Evaluation*	*Utilising a Variety of Strategies to Assess Student Learning*	*‘The method and contents of teaching in case study conference conducted in the replacement of face-to-face hands-on training are likely to help carry out the practicum’.*
*Leadership and Professionalism*	*Insisting Urgent Regulation Preparation for Uncertain Situations*	*‘I hope our academic society will ask the “Korean Accreditation Board of Nursing Education” to come up with measures to prepare for this situation’.*
		*‘We need a way for both professors and students to operate and participate in nursing practicum without anxieties’.*
		*‘I have personally inquired with the “Korean Accreditation Board of Nursing Education” several times about how to prepare a nursing practicum for this situation’.*

## Discussion

The workshop satisfaction regarding the teaching methods applied in virtual reality for nursing faculties was high, and the faculty members’ attitude and knowledge on education using ICT were improved through the workshop. The results of the content analysis revealed that engagement, application of digital technology to the curriculum, pedagogy, management of the learning environment, and faculty professional learning were important elements corresponding to PDC. Theories and principles of adult learning, curriculum design and implementation, nursing practice, communication, collaboration and partnership, monitoring and evaluation, and leadership and professionalism were identified as important elements corresponding to nurse educator core competencies in the conceptual framework.

Enhancing the faculty’s proficiency in utilizing technology effectively, selecting and modifying digital resources for educational purposes, facilitating learner engagement with ICT, and establishing appropriate evaluation mechanisms for digital learning is crucial [11]. It is essential to identify ICT resources [11, 12] that can serve as mechanism for educational purposes. Identifying and understanding the available resources is the first step toward conducting education using ICT, as participants in the program developed in this study described it as a valuable opportunity to understand virtual space and obtain information about online platforms and resources. Applying digital technology to education requires careful consideration of learners’ engagement, teaching methods, and evaluation [13]. Participants in this study expressed that they learned strategies to apply ICT to education through the experience of a series of hands-on processes and the introduction of actual cases during the workshop. Many of the faculty training programs implemented in previous studies have also been reported to provide participants with opportunities to practice and receive feedback [14–16]. However, since this was a once-off workshop without follow-up, the faculty members received feedback on a series of hands-on processes, but they could not share their experiences regarding implementing the new strategies in their curriculum.

In addition, as nursing science is a practical discipline, the practice curriculum to foster engagement for undergraduate students are very important. Through nursing practice such as clinical practice, simulation, and case studies, students develop clinical judgment, clinical reasoning, and critical thinking skills by applying the theories they learned to nursing practice [17, 18]. It is important to design an effective course of practice to determine which technical devices can maximize education outcomes [19] and what elements can be linked to clinical sites. Indeed, the findings from this workshop highlight that instructors were able to find strategies to apply to nursing practice.

Overall, the participants were satisfied with the workshop and evaluated the content as meeting the needs of providing well-structured educational strategies for disaster situations such as the COVID-19 pandemic. In the education for empowerment of faculties, meeting the faculties’ learning needs is the most important part of engaging participation [14]. Additionally, the participants described that through this workshop, they learned about various resources for the use of digital technologies in education and felt confident in using effective educational strategies with various technology resources. According to the framework for building digital competency proposed by UNESCO [16], the training workshop conducted in this study is considered to be a program that achieved the level for knowledge acquisition and partially achieved the knowledge deepening level. The level of knowledge creation where facilitators apply ICT to education may require more than a one-off interactive session.

Nurse educators need to enhance their proficiency in utilizing technology effectively because they play a critical role in promoting the acquisition of digital competences among nursing students. Considering that contemporary nurses need digital competencies to integrate new technology into their practice, digital competencies of nursing educators are important but previous studies have suggested lack of digital knowledge and skills among nursing educators [20]. Nursing faculty should incorporate ICT competencies into the nursing education program. Through educational initiatives, learners should be cultivated to enhance their abilities in acquiring knowledge, skills, and competencies related to effective communication, cooperation, and partnership, utilizing ICT competency. Additionally, an evaluation of learning outcomes and the competencies emerging during the educational process should be conducted when the integrated program is implemented.

In pandemic situations such as COVID-19, MERS, and SARS faculties need to utilise ICT to enable distance education for students. Preparing teaching methods to be applied to new technologies related to the virtual world can be burdensome due to consideration of various educational factors [21]. However, to provide education using ICT, faculty members should have basic skills and technical knowledge [7]. Technology needs to be seen as a means for the achieving objectives to be achieved, not as a focus itself.

## Conclusions

The findings of the study show that it is critical importance of integrating both digital proficiency and nursing practice education to enhance nursing faculties’ digital teaching competence. Considering the present urgent need to maintain social distancing across all economic sectors, preparing teaching methods by applying new technologies can help nursing faculties to effectively educate distance learners in pandemic situations such as the COVID-19, MERS, and SARS. The nursing faculty development program for digital teaching competency provides nursing faculties with self-confidence and essential skills to teach students using virtual and online simulation. Further studies are warranted to determine the optimal methods and composition of the faculty development program.

## Data Availability

All data set and materials are available from the corresponding author.

## Abbreviations

AI: Artificial Intelligence
COVID-19: Coronavirus Disease
ICT: Information and Communication Technology
IT: Information Technology
MERS: Middle East respiratory syndrome coronavirus
PC: Personal Computer
PDC: Pedagogical Digital Competence
SARS: Severe Acute Respiratory Syndrome
TBL: Team-based Learning
TOT: Train-the-Trainer (TOT)
UNESCO: United Nations Educational, Scientific, and Cultural Organisation
XX: X University
